# Intra-individual variability in animal models of bipolar disorder

**DOI:** 10.1186/s40345-019-0144-1

**Published:** 2019-04-02

**Authors:** Emily R. Hawken, Elisa Brietzke, Claudio N. Soares

**Affiliations:** 1 Department of Psychiatry, Providence Care Hospital, Queen’s University School of Medicine, 752 King Street West, Postal Bag 603, Kingston, ON K7L7X3 Canada; 2 The Canadian Biomarker Integration Network in Depression (CAN-BIND), Toronto, ON Canada

Dear Editor,

We read with interest the outstanding manuscript from Beyer and Freund (2017) published in International Journal of Bipolar Disorder, summarizing the literature on animal models for Bipolar Disorder (BD). Putative pathways involved both in mania and depressive phases of the disease were described and discussed in detail (e.g. stress sensitivity, dopaminergic abnormalities, immune activation, circadian changes) including the scope of these pathways and the extent to which they are intertwined. The authors also raised a point which is, in our opinion, a major conundrum in the current research on animal models for BD: the lack of paradigms that could mimic the phasic variation of behaviors that are observed in humans and represent the main characteristic of this disorder. The discovery of the mechanisms underlying the transition between euthymia to a mood episode (depression or mania/hypomania) could have a remarkable impact on the development of new approaches to reduce recurrence, long term clinical deterioration, and neuroprogression (Kessing and Andersen 2017; Berk et al. 2011).

As Beyer and Freund (2017) pointed out, one of the most promising hypotheses for the pathophysiology of BD is the dopamine hypothesis. Dysfunction in dopaminergic transmission is often seen during manic episodes associated with hyperdopaminergia as well as in depressive episodes with hypodopaminergia (Berk et al. 2007). The same investigators had previously developed an intriguing dopamine-centric BD animal model which relies on the introduction of DNA fragments into cells of specific parts of the brain via a viral vector (Freund et al. 2016). This procedure allows cells (e.g., glutamatergic neurons) to over-express D1 receptor (D1R). Over-expression of D1R in the medial prefrontal cortex of rats promotes manic-like behaviors. Conversely, termination of viral over-expression precipitates a depressive-like set of behaviors (Freund et al. 2016). Other models have attempted and failed to emulate the cyclic, intra-individual high-variability nature of BD.

One could argue that existing animal models for BD lack an accurate quantification of intra-individual variability in behavior; this would be paramount to enhance its utility in clinical research, and advance our understanding of the underlying neurobiology associated with relapse and/or recurrence.

Recent developments in behavior-tracking technologies and big data could revolutionize translational research particularly in the behavioral sciences. For instance, digital phenotyping (via ecological momentary assessments) across species may aid in substantiating the face, predictive, and construct validity of BD animal models. Behavioral signatures of the BD phenotype in animal models could more readily elucidate brain mechanisms of this condition; likewise, a valid digital behavioral fingerprint of BD in humans will elucidate ecological correlates (behavioral, environmental) that contribute to disease cyclicity. Continuous and longitudinal data capture is likely to be an essential step towards a better characterization of such a heterogeneous illness trajectory like that seen in BD. Furthermore, informatics and computational neuroscience will be critical for translation, linking behavioral endpoints across species.

Naturally, intra-individual variability in expression of symptoms is present in all psychiatric disorders, and translating this characteristic to animal models remains a significant challenge. However, in other disorders intra-individual changes are only one aspect of the expression of psychopathology, yet in bipolar disorder the alternate expression of dramatically different sets of symptoms is the core clinical (mental, cognitive, and behavioral) manifestation. Different from other psychopathologies, intra-individual variability in BD happens in two dimensions: recurrence and polarity, and potentially in a third, clinical progression, adding even more complexity to translational research. Typically, animal models of mental health disorders (i.e., schizophrenia, depression, etc.) only model sypmtoms in a single dimension, i.e., recurrence. Thus, innovative animal models and behavioral assays may constitute an opportunity for translational research to advance our understanding of BD disease progression and ultimately improve the lives of those suffering from this disabling condition.

## References

[CR1] Berk M, Dodd S, Kauer-Sant’anna M, Malhi GS, Bourin M, Kapczinski F, Norman T (2007). Dopamine dysregulation syndrome: implications for a dopamine hypothesis of bipolar disorder. Acta Psychiatr Scand Suppl.

[CR2] Berk M, Kapczinski F, Andreazza AC, Dean OM, Giorlando F, Maes M, Yücel M, Gama CS, Dodd S, Dean B, Magalhães PV, Amminger P, McGorry P, Malhi GS (2011). Pathways underlying neuroprogression in bipolar disorder: focus on inflammation, oxidative stress and neurotrophic factors. Neurosci Biobehav Rev.

[CR3] Beyer DKE, Freund N (2017). Animal models for bipolar disorder: from bedside to the cage. Int J Bipolar Disord..

[CR4] Freund N, Thompson BS, Sonntag K, Meda S, Andersen SL (2016). When the party is over: depressive-like states in rats following termination of cortical D1 receptor overexpression. Psychopharmacology.

[CR5] Kessing LV, Andersen PK (2017). Evidence for clinical progression of unipolar and bipolar disorders. Acta Psychiatr Scand.

